# Development of a generic focal spot measurement method suitable for bore‐type linacs

**DOI:** 10.1002/acm2.70077

**Published:** 2025-04-07

**Authors:** Hidetoshi Shimizu, Kazuharu Nishitani, Tomoki Kitagawa, Koji Sasaki, Takahiro Aoyama, Takeshi Kodaira

**Affiliations:** ^1^ Department of Radiation Oncology Aichi Cancer Center Hospital Nagoya Aichi Japan; ^2^ Division of Medical Physics School of Medical Sciences Fujita Health University Toyoake Aichi Japan; ^3^ Precise Accuracy Laboratory Tokyo Japan; ^4^ Graduate School of Radiological Technology Gunma Prefectural College of Health Sciences Maebashi Gunma Japan

**Keywords:** focal spot position, quality assurance, Radixact, tomotherapy

## Abstract

**Purpose:**

In linear accelerators, deviations in the x‐ray focal spot position significantly affect the accuracy of radiation therapy. However, as the focal spot position in bore‐type linac systems such as the Radixact system, cannot be assessed using conventional methods, a new evaluation method is required. This study aimed to develop a novel method to measure the focal spot position of Radixact and evaluate any deviations from the ideal x‐ray focal spot position.

**Methods:**

A structurally simplified measurement system was developed to evaluate the focal spot position of the Radixact system. This system consisted of a vertically aligned metal bar and an ionization chamber, which was moved stepwise to acquire the beam profiles. The focal spot position deviation was calculated based on the center differences of the profiles obtained from two different upstream and downstream locations of the metal bar.

**Results:**

The measurement results indicated that the focal spot position shift was 0.42 mm and −0.36 mm at the target height in the IEC‐X and ‐Y directions, respectively. The measurement uncertainty was 0.187 mm, confirming a slight deviation from the ideal focal position.

**Conclusions:**

This study developed a novel method to accurately evaluate the x‐ray focal spot position of the Radixact system, which can potentially be applied to other conventional linear accelerators and bore‐type systems, such as Halcyon, to improve the accuracy of radiotherapy. However, its generalizability and applicability to different radiotherapy machines must be explored further.

## 1 INTRODUCTION

In medical linear accelerators, the ideal position of the x‐ray focal spot is vertically above the collimator rotation axis and the gantry rotation center when the gantry angle is 0°. In addition, the acceleration guide of some medical linear accelerators is positioned vertically, and the electron beam must pass precisely through the center of the guide. Proper alignment of the focal spot position is required to converge the radiation isocenter onto the mechanical isocenter, center the beam accurately, and maintain a symmetric beam profile. The focal spot position is critical to ensure the accuracy of Monte Carlo modeling^1^, ^2^ and determine the limits of the modulation transfer function (MTF) for imaging.^3^, ^4^, ^5^, ^6^ However, this position is usually not evaluated as it is challenging to do so. Furthermore, this practice has not yet been incorporated into the guidelines established by the American Association of Physicists in Medicine (AAPM) Task Group report.^7^, ^8^ In clinical settings, deviations in the focal spot position cause the collimator angle dependence of the wedge coefficient in typical radiotherapy treatment machines^9^ and alter the proper patient alignment in image‐guided radiation therapy (IGRT), including unique radiotherapy treatment machines such as the Radixact system, featuring a kV source. Therefore, a method to accurately evaluate the focal spot position is urgently required.

Several reports have evaluated the focal spot positions of conventional medical linear accelerators. Lutz et al. developed a method using a punch aperture fixed to the collimator assembly to determine the intersection of the collimator rotation axis with the film pack on a couch.^10^ Recently, more convenient techniques have emerged. Jyiri et al. proposed two methods to measure the focal spot position.^11^ The first method involved mounting a jig for the collimator assembly with an ionization chamber near the 50% beam edge and using the signal generated by the collimator rotation. The second method used a rod phantom placed at different distances from the source and utilized images taken at various collimator angles. Chojnowski et al. presented a phantomless method that exploits the height differences between the multi‐leaf collimator (MLC) and jaws using the electronic portal imaging device (EPID).^12^, ^13^ Slama et al. developed a method by installing a special fixture with ball bearings at the top and bottom of the gantry to identify the focal spot position.^14^ However, these methods cannot be applied on the Radixact system, which is a bore‐type linac, because it lacks an EPID and the necessary space to mount a jig. Therefore, we developed a new method to measure the focal spot position of Radixact and evaluate any deviations from the ideal x‐ray focal spot position.

## 2 METHODS

### 2.1 The Radixact system

This system is a treatment machine using helical tomotherapy,^15^ which was the basic concept introduced in 1993.^16^ On the opposite facet of the 6 MV linear accelerator, there are megavoltage (MV) image detectors to provide image‐guided radiotherapy (IGRT) using megavoltage CT (MVCT) technology. The source‐axis distance is 85 cm, as opposed to 100 cm for the conventional linacs. The radiation field is defined by the MLCs, which have a width of 6.25 mm at the isocenter in the International Electrotechnical Commission (IEC)‐X direction and cover 40 cm. Meanwhile, the IEC‐Y direction is controlled by a jaw whose width can vary between 1, 2.5, and 5 cm. Due to system limitations, the collimator remains stationary while the gantry rotates continuously.

### 2.2 Theory

**FIGURE 1:**
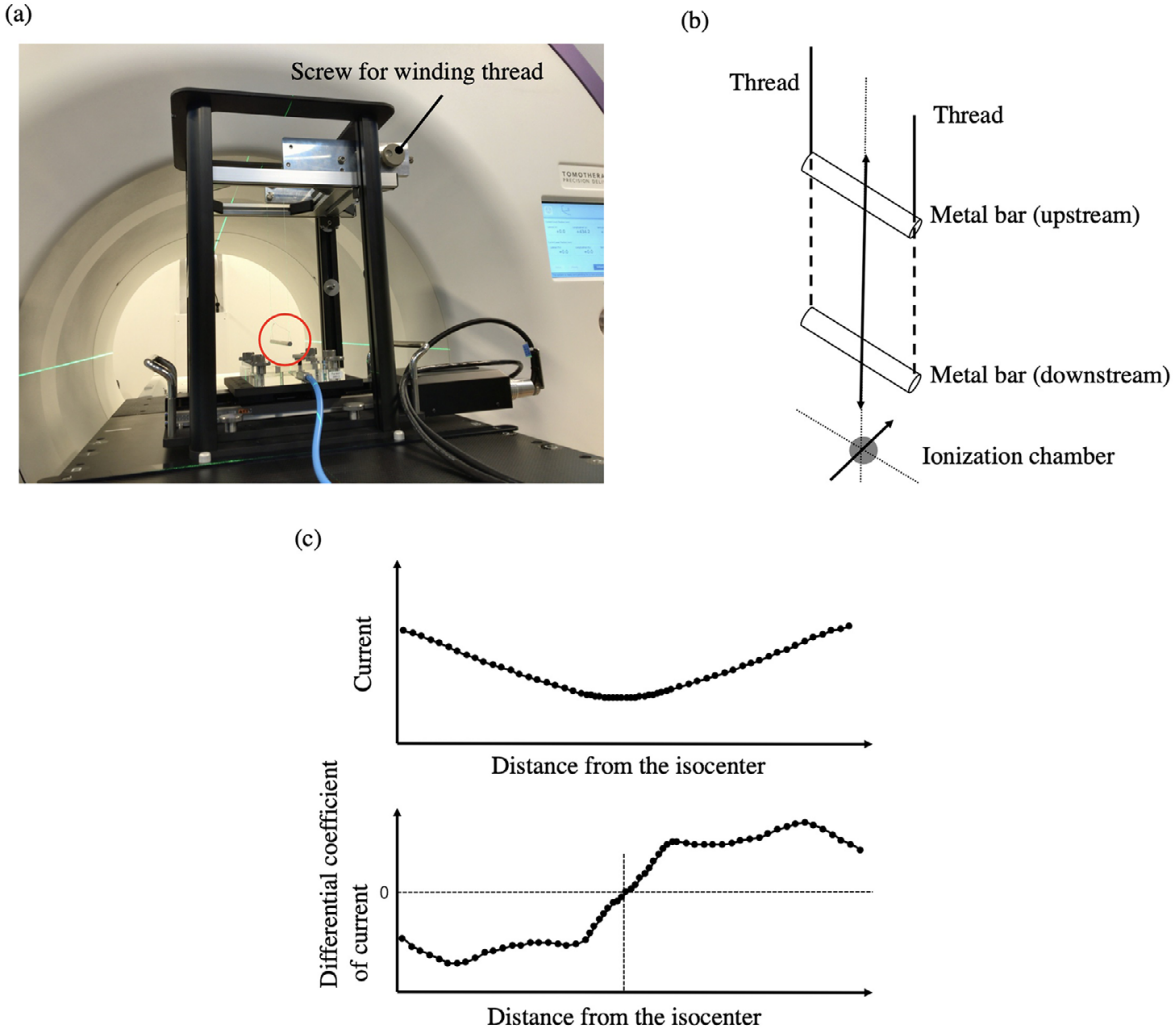
System consists of a metal bar suspended from a thread. Figure (a) shows a metal bar (in red circles) stopping downstream. By winding the thread with a screw, the position of the metal bar can be moved, as shown in the schematic diagram (b). Below the metal bar is a stage that moves the ionization chamber along its short axis. The top panel of (c) shows an example of the profile after passing through the metal bar. The bottom panel of (c) illustrates the profile of the differential coefficient of current per distance from the isocenter.

**FIGURE 2:**
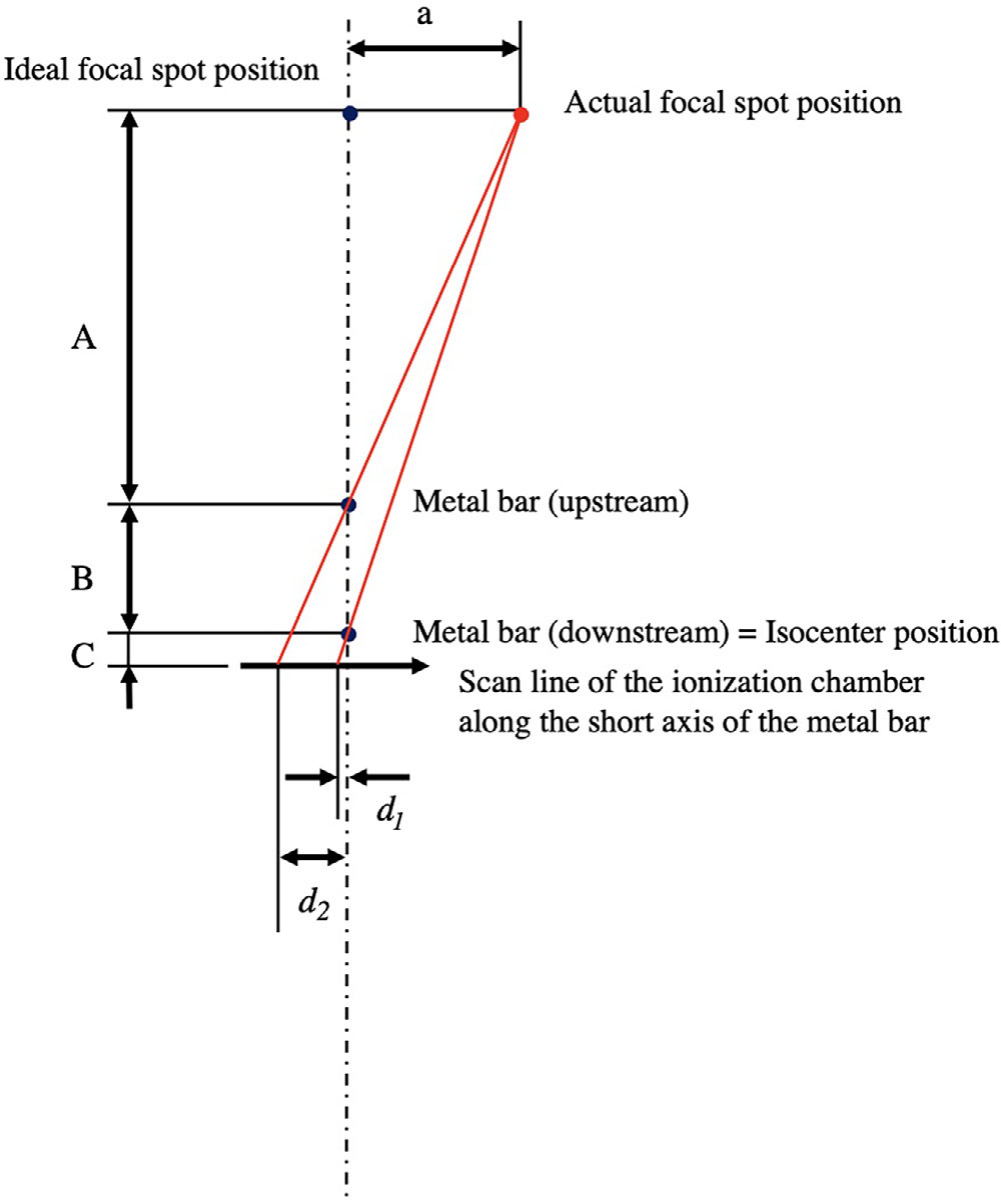
Representation of the arrangement diagram for measuring the focal spot position. *d*_1_ and *d*_2_ represent the deviation of the center of the dip from the MVCT center at the upstream and downstream positions, respectively. A, B, and C represent the distances from the upstream metal bar to the focal spot position, between metal bars, and from the downstream metal bar to the profile acquisition surface, respectively. Abbreviation; MVCT, megavoltage CT.

The collimator cannot be rotated in Radixact, so the ideal focal spot position is vertically above the gantry rotation center when the gantry angle is 0°. In addition, the acceleration guide is positioned vertically, and the electron beam must pass precisely through the center of the guide. As identifying the gantry rotation center is difficult, we substituted it with the MVCT image center in this study. When using instruments such as a treatment couch or water phantom for measurement, it is difficult to accurately evaluate the focal spot position in the vertical direction due to mechanical and operational constraints. While a laser level can be used to guide the vertical direction, there is uncertainty due to visual inspection. Therefore, we developed a system in which a metal bar (length: 14.5 cm, diameter: 0.5 cm) is suspended with a thread. The center difference of the profiles passing through the upstream and downstream metal bars was calculated. Then, the magnification was corrected to calculate the deviation at the focal position (Figure 1). The position of the metal bar can be adjusted in the vertical direction by winding the thread using a roller. The number of rotations of the roller determines the movement of the bar, which shifts 20.473 cm with two rotations. The ionization chamber was placed on a stage in the lower part of this system. This chamber moved stepwise during irradiation, and the resulting current was recorded with an electrometer. The profiles were obtained by outputting the current value relative to each ionization chamber position. The top panel of Figure 1c shows an example of the profile after passing through the metal bar. We differentiated the current per distance from the isocenter, as shown in the bottom panel of Figure 1c, and defined the position where the differential coefficient becomes zero as the center of the profile. Figure 2 shows a diagram for determining the focal spot position. The deviation of the focal spot position (a) can be calculated using Equation (1).

(1) $$a=\frac{d_{2-}d_{1}}{\frac{B+C}{A}-\frac{C}{A+B}}$$

Here, *d*_1_ and *d*_2_ represent the deviation of the center of the dip from the MVCT center at the upstream and downstream positions, respectively. A, B, and C represent the distance from the upstream metal bar to the focal spot position, the distance between the metal bars, and the distance from the downstream metal bar to the profile acquisition surface. If the distance from the focal position to the downstream position (A plus B) is 85 cm, B is 20.473 cm, and C is 3.65 cm, then Equation (1) can be rewritten as Equation (2) based on numerical calculations using A, B, and C.

(2) $$a=\frac{d_{2-}d_{1}}{0.33}$$

### 2.3 Procedure

The tests were conducted on the Radixact system at Aichi Cancer Center Hospital. After aligning the downstream metal bar using a green laser line indicating the isocenter, the MVCT was performed. Based on the image registration result, the metal bar was aligned with the MVCT center by shifting the couch. The MVCT was retaken to confirm that the metal bar was at the center. Subsequently, the beam was delivered with the fixed field of 5 cm (IEC‐Y direction) × 10 cm (IEC‐X direction) at the isocenter, and the profile was obtained by moving the ionization chamber in the IEC‐X direction stepwise on the stage. Next, the metal bar was moved upstream in the vertical direction by winding up the thread holding it with a roller to obtain the profile similarly. Before measuring, we visually confirmed that the metal bar had stopped. TM30013 (PTW) and UNIDOS (PTW, Range: Middle) were used as the ionization chamber and the electrometer, respectively. For the measurements, 15 mm PMMA was used as the build‐up material. Table 1 displays the measurement intervals for each distance from the isocenter of the ionization chamber. The delay for measurement in each step measurement was 3 s, and the sampling number was 5. The gantry angle was set to 0° (based on the installation concept of the Radixact, the plate level installed opposite the linac is 0°), and measurements were started when the metal bar stopped swinging. We also performed similar measurements in the IEC‐Y direction.

**TABLE 1:** Measurement intervals for each distance from the isocenter using an ionization chamber.

Distance from the isocenter [mm]	Measurement interval [mm]
−10 to −5	1
−5 to −1	0.2
−1 to 1	0.1
1 to 5	0.2
5 to 10	1

To calculate the uncertainty in the measurements, repeatability was evaluated by 10 consecutive measurements and reproducibility by three measurements at one‐week intervals.

## 3 RESULTS

### 3.1 Displacement of the focal point position

**FIGURE 3:**
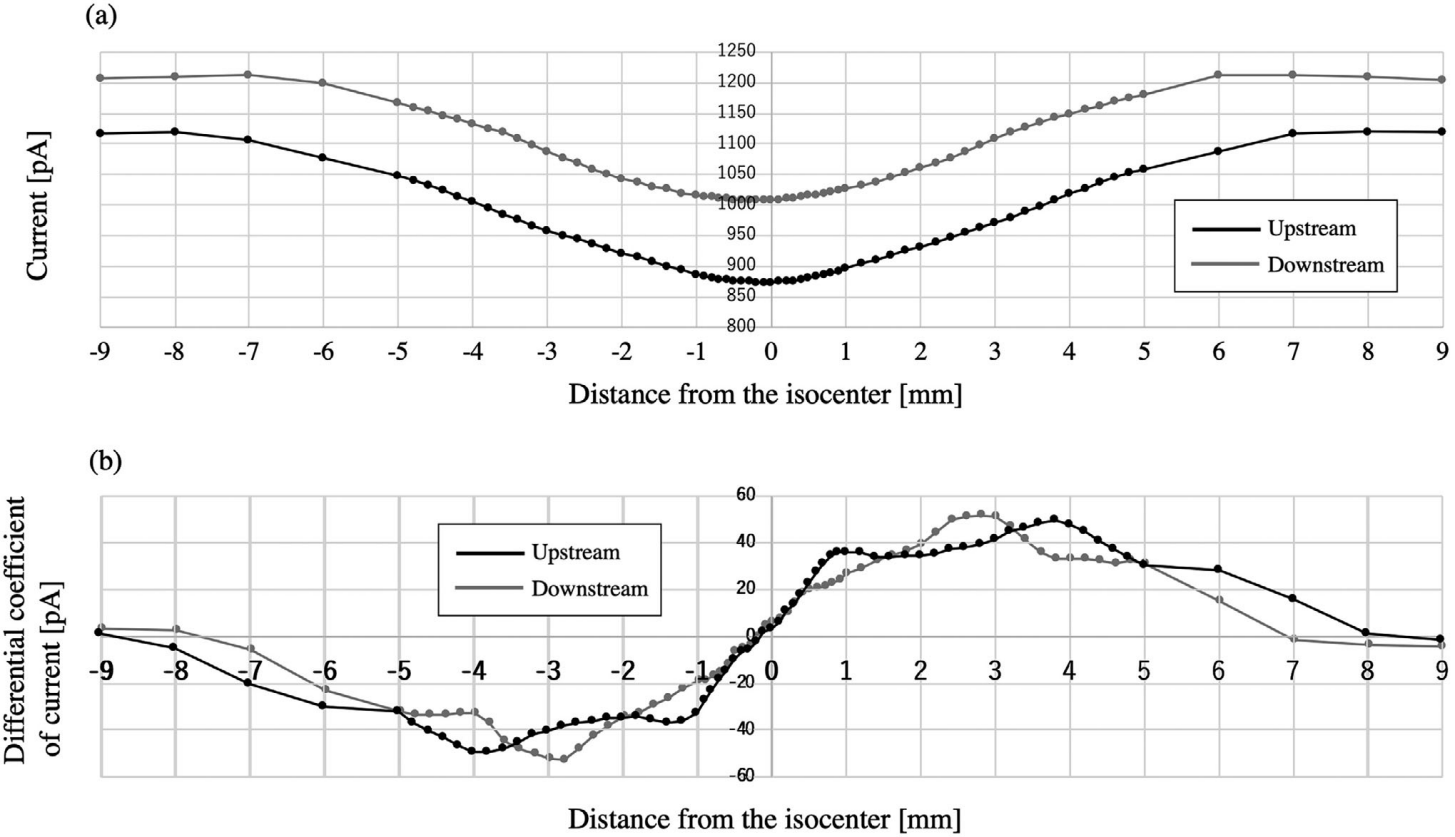
Focal point deviation in the IEC‐X direction. (a) shows the profile obtained through the ionization chambers at the upstream and downstream locations (indicated in black and gray, respectively). The metal bars reduced the current in the center of the profile. (b) was obtained by calculating the differential coefficient of these profiles. Abbreviation; IEC‐X, International Electrotechnical Commission‐X direction.

**FIGURE 4:**
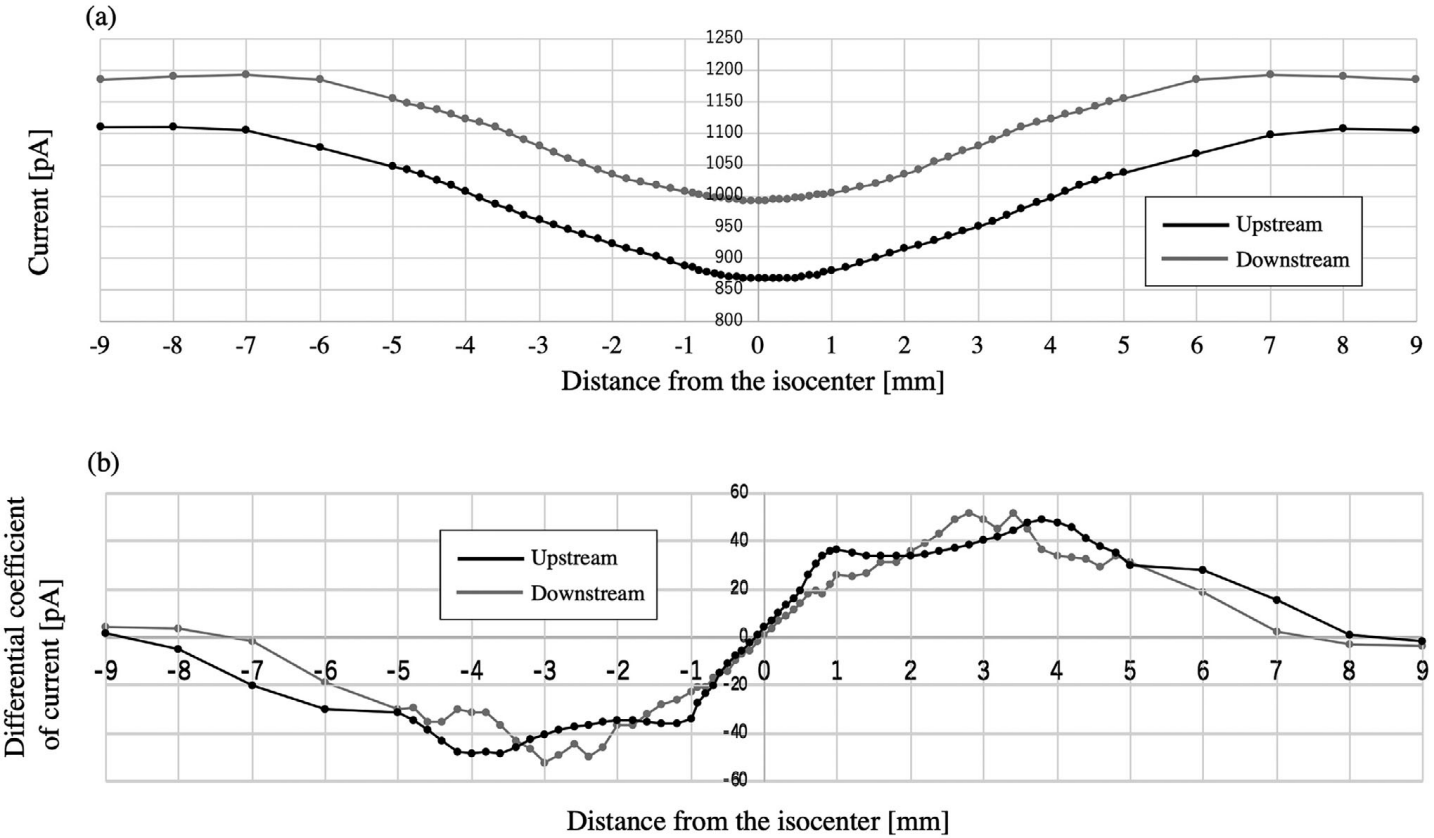
Focal point deviation in the IEC‐Y direction. (a) shows the profile obtained through the ionization chambers at the upstream and downstream locations (shown in black and gray, respectively). (b) was obtained by calculating the differential coefficient of these profiles. Abbreviation; IEC‐Y, International Electrotechnical Commission‐Y direction.

Figure 3a shows the profile obtained through the ionization chambers at the upstream and downstream locations in the IEC‐X direction. The metal bar reduced the current at the center of the profile. Figure 3b was obtained by differentiating the current per distance from the isocenter. Similar measurements were performed in the IEC‐Y direction (Figures 4a, b).

The values of x at *y* = 0 for the differentiated upstream or downstream profiles, namely *d*_1_ and *d*_2_, were −0.14 ± 0.01 mm and 0.00 ± 0.01, respectively, in the IEC‐X direction, or −0.14 ± 0.02 mm and –0.02 ± 0.01 mm in the IEC‐Y direction. The displacement of the focal point position calculated based on Equation (2) was 0.42 mm and −0.36 mm for the IEC‐X and IEC‐Y directions, respectively.

The time from setting up the phantom to completing the measurement of the focal position in the IEC‐X and Y directions was approximately 90 min.

### 3.2 Uncertainty budget

Table 2 summarizes the uncertainty budgets of the measured focal spot position. The repeatability and reproducibility in the measured values between upstream and downstream can be classified as Type A and estimated from their respective standard deviations. The minimum measurement interval of 0.1 mm was included as the uncertainty in the measured values categorized as Type B. As the uncertainties of A, B, and C are much smaller (less than 1/10) than those of *d*_1_ and *d*_2_, they were excluded from the uncertainty calculation.^17^ Finally, the combined uncertainty of the measured focal spot position was 0.187 mm.

**TABLE 2:** Uncertainty budgets of the measured focal spot position.

Quantity source of uncertainty	Uncertainty type	Standard uncertainty [mm]
*d*_1_ (repeatability)	Type A	0.048
*d*_2_ (repeatability)	Type A	0.048
*d*_1_ (reproducibility)	Type A	0.087
*d*_2_ (reproducibility)	Type A	0.087
*d*_1_ (measurement interval:0.1 mm)	Type B	0.087
*d2* (measurement interval:0.1 mm)	Type B	0.087
Combined standard uncertainty		0.187

## 4 DISCUSSION

Here, we developed a new method to measure the x‐ray focal point position of the Radixact system and to evaluate any deviations of the actual focal point from the ideal one. Resultantly, the focal point position was found to be shifted by 0.42 mm and −0.36 mm in the IEC‐X and ‐Y directions, respectively. Conventional methods cannot be used to measure the focal point of the Radixact system, a bore‐type linear accelerator, due to the lack of space for mounting the EPID and fixture and the inability to rotate the collimator. This study is the first to report the focal spot measurement of a bore‐type linac.

Since the focal spot position influences geometric accuracy, including beam flatness and symmetry, as well as the size and location of radiation isocenters, optimizing the focal spot position is of great clinical significance. In other words, measuring and calibrating the focal spot position at periodic cycles is expected to improve the radiotherapy accuracy.

The measurement of the focal spot position has not been widely adopted because the measurement is complicated and time‐consuming.^12^, ^14^ Although our system solves the complexity problem, the measurement time is still longer, taking approximately 90 minutes for the combined measurements of the IEC‐X and Y directions. In the future, it will be necessary to automate the process from setting up the phantom to measuring it as possible and shortening the measurement time. On the other hand, measuring it is essential, if infrequently, because the focal spot position is the basis of geometric accuracy.

In Radixact, the target is in the vacuum of the linac. As the accelerator does not have a steering coil, the linac position is adjusted manually by tightening the bolts that secure it so that the beam center is at the center of the MLC in the IEC‐X direction, and the jaw is at the center in the IEC‐Y direction. These adjustments are checked to be within tolerable limits using the “x‐alignment of source” and “y‐jaw centering” tests during commissioning and periodic QA.^16^ However, if the MLC or jaw is misaligned minutely offset from the beam center, even within tolerable limits, these errors can affect the absolute position of the linac. Our method can circumvent this problem. Specifically, after the focal position is measured and the linac is placed in the exact position, the MLC or jaw can be verified to be within tolerance with respect to the linac, thereby enabling more accurate component positioning. However, adjusting the focal spot position could have a significant effect on the beam. Therefore, we have not confirmed whether this adjustment process works well for the treatment machine used in clinical practice in our facility. In addition, while the jaw can be moved electronically, the MLC unit is a physically fixed mechanism and does not have an electronic movement function. Such structural constraints might limit the adjustment of the components to the ideal focal spot position.

This method can be applied to conventional linacs, as well as Radixact, Halcyon, and other bore types. The developed system structure is simple and easy to replicate with few materials.

There are some limitations to this study. First, the method is specific to a particular device (Radixact) and has not been applied to other devices. Further study is needed for generalization, especially since different error factors may exist for conventional linacs. In addition, measurements were made on a Radixact system at a single facility and have not been verified for reproducibility at other facilities or instruments. To enhance the value of this study, it will be essential to evaluate its reproducibility at other facilities in the future.

## 5 CONCLUSIONS

In this study, we developed a novel method to evaluate the x‐ray focal spot position on the Radixact system. Using a simple system of metal bars and an ionization chamber, we could accurately measure the focal spot deviations. This method can be applied to other bore‐type systems, such as Halcyon, as well as conventional linear accelerators. However, further research and clinical applications are needed to explore this method for different radiotherapy machines.

## AUTHOR CONTRIBUTIONS

Hidetoshi Shimizu and Kazuharu Nishitani conceived the study, analyzed and interpreted the data and drafted the manuscript. Tomoki Kitagawa, Koji Sasaki, Takahiro Aoyama, and Takeshi Kodaira were involved in the study design and significantly contributed to the editing of the manuscript. All authors read and approved the final manuscript.

## CONFLICT OF INTEREST STATEMENT

The authors declare no conflicts of interest.
